# Coinfection with *Strongyloides* and *Ascaris* in a COVID-19-positive male presenting with acute abdomen: a case report

**DOI:** 10.2217/fmb-2022-0027

**Published:** 2022-07-28

**Authors:** Sweta Singh, Uday S Singh

**Affiliations:** ^1^Department of Microbiology, Autonomous State Medical College, Mirzapur UP, India; ^2^Department of Radiodiagnosis, Advance Diagnostic Centre, Lucknow, UP, India

**Keywords:** antihelminthic, *ascaris*, coinfection, COVID, *strongyloides*

## Abstract

*Ascaris lumbricoides* and *Strongyloides stercoralis* are soil-transmitted helminthic infections usually seen in people with poor socioeconomic conditions, hygiene and fecal sanitation living in endemic countries. Here, we present a case of coinfection in a COVID-positive older adult male presenting to our facility with symptoms of acute abdomen. Investigative workup guided timely diagnosis of the case. Prompt initiation of antihelminthic drugs together with antibiotics/antivirals for COVID symptoms resulted in favorable outcome in the case. A high index of suspicion on the part of the treating and diagnosing doctor is required in the COVID era. This will help not only in diagnosis but will also give an understanding to the exact pathogenesis for better patient outcome.

Helminthic infestation is common among people living in tropical and subtropical areas. *Ascaris*, *Strongyloides*, *ancylostoma* and *trichuris*, among others, are some of the more common helminths worldwide. Their prevalence is usually related to poverty and poor socioeconomic status, hygiene and fecal sanitation.

Infection with *Ascaris lumbricoides* starts with the ingestion of embryonated eggs that hatch into larva and pierce the duodenal wall to reach the portal circulation; via the liver, it migrates to the lungs and evolves into more advanced stage larva. Larvae of *Ascaris* then reach the alveoli of lungs, migrate to the upper respiratory tract; with the sputum, they are swallowed back into the intestines. In the small bowel, they develop into adults, and usually the patient remains asymptomatic. Intestinal obstruction is usually seen, with a high worm load and, if left untreated, can also result in intestinal perforation. These two conditions form the differential diagnosis of acute abdomen in humans, especially children with poor feco-oral hygiene. Hepatic conditions associated with *Ascariasis* are usually seen when the worm enters the ampulla of Vater and usually includes biliary colic, acute cholangitis, acute cholecystitis, acute pancreatitis and hepatic abscess, among other conditions [1].

*Strongyloides stercoralis* is a soil-transmitted nematode; the filariform larvae enter the human host via skin penetration at the feet area or mucous membrane penetration via ingestion of raw contaminated vegetables and herbs. The larvae then enter the blood stream and migrate further to lungs and alveoli swallowed back to the intestines. Here, the larvae mature into adult females and lay their eggs on the gastrointestinal mucosa. These eggs hatch into rhabditiform larvae and are excreted in feces. These develop into filariform larvae in soil, and hence the life cycle continues. In immunocompetent individuals, infection usually remains asymptomatic, or transient diarrhea and abdominal pain may be seen. The autoinfective cycle is seen in this nematode as some larvae may remain in the gastrointestinal tract, and when the host is immunocompromised, they change into infective filariform larvae to penetrate the intestinal mucosa and continue the pulmonary life cycle. In this way, they give rise to hyperinfection syndrome and disseminated disease in an immunocompromised host [2].

Here, we report an interesting and rare case of coinfection with *Ascaris* and *Strongyloides* in a COVID-positive older adult male presenting with acute abdomen in our emergency department.

## Case report

A 58-year-old male presented to the emergency department of our institution with chief complaints of breathlessness, dyspnea and abdominal pain for the past 4 days. The abdominal pain was acute colicky in nature, periumbilical, not radiating to other sites and associated with abdominal upset with vomiting. The patient recalled the presence of fever, sore throat, dyspnea and gastric upset with upper respiratory tract symptoms from previous 14 days. He had been diagnosed with diabetes 8 years prior and was on daily insulin injections. The patient was diagnosed with rheumatoid arthritis 2 years earlier and was prescribed oral methylprednisolone for pain and swelling in the joints, initially 40 mg in two divided doses and later tapered to 20 mg once daily, followed by 10 mg daily. Due to the ongoing COVID pandemic, he was advised to test for infection; naso- and oropharyngeal swabs were obtained and sent for RT-PCR testing for COVID-19. The test was positive, and treatment was initiated with injectables including amphotericin B (for antifungal prophylaxis), aztreonam and oral methylprednisolone with random blood sugar monitoring.

**Figure 1.**
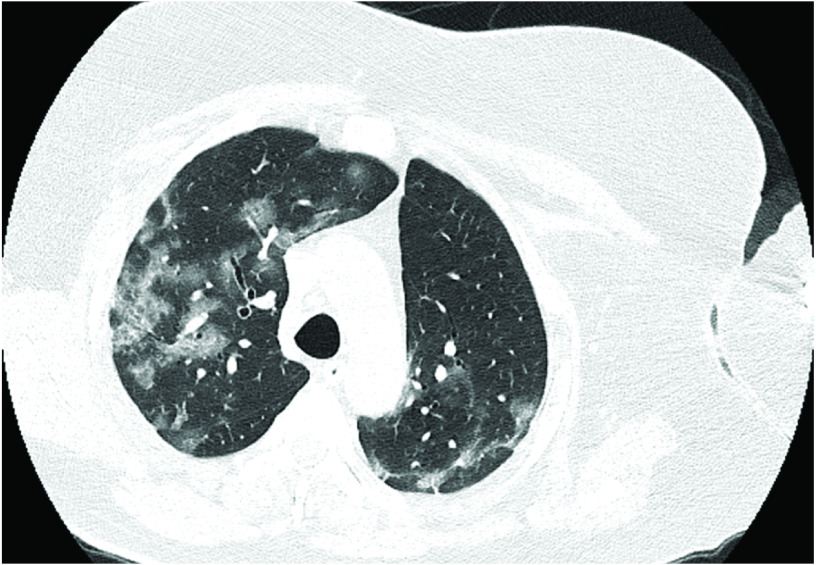
High-resolution CT scan lung showing ground glass opacities in a COVID-positive male patient.

**Figure 2.**
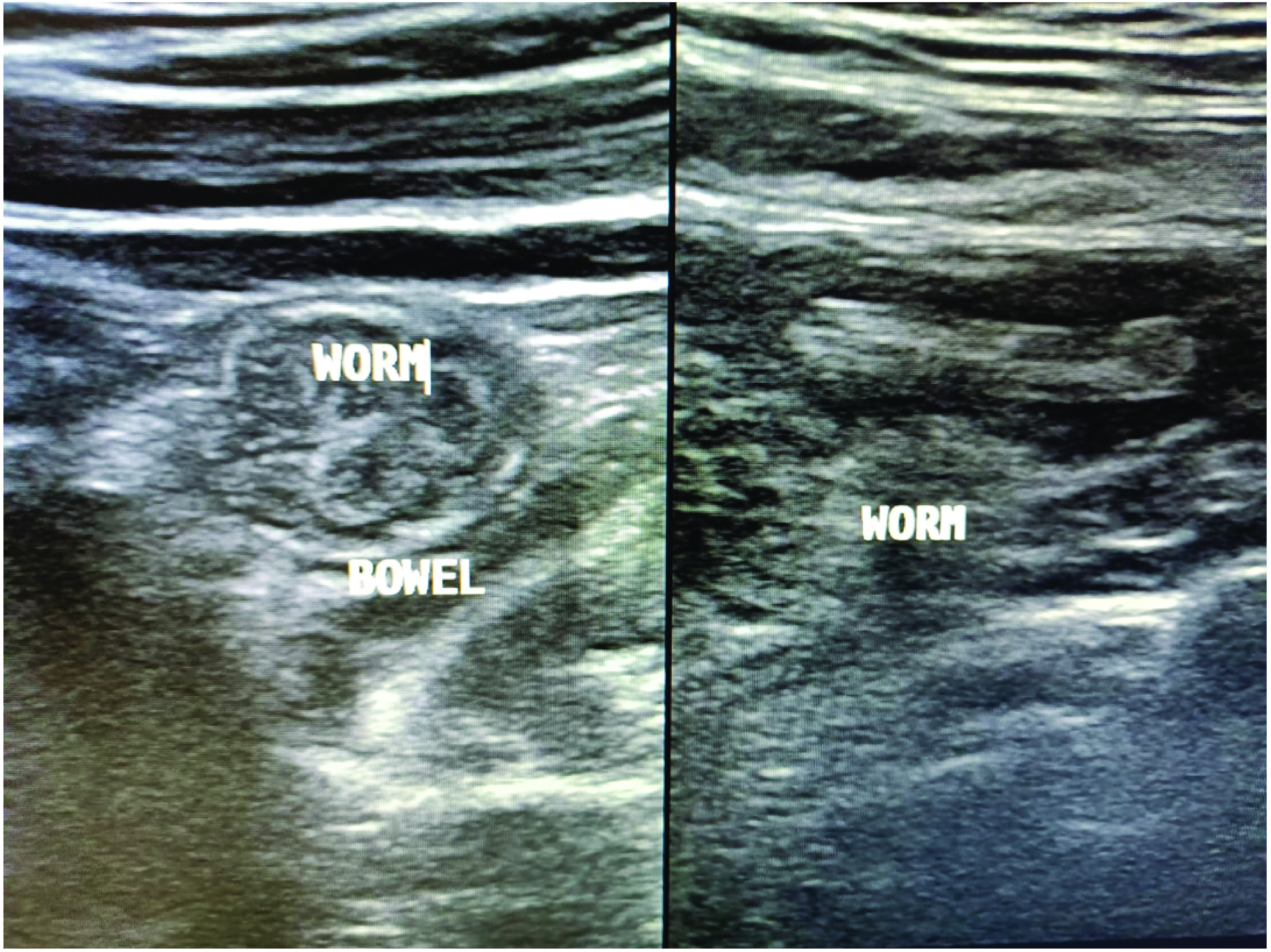
Abdominal ultrasound showing multiple tubular echogenic structures resembling *Ascaris lumbricoides*.

Routine blood investigations revealed lymphopenia, raised C-reactive protein and procalcitonin levels as well as raised serum amylase levels. Eosinophil absolute count (EAC) increased to >660 cells/ul on the fifth day of admission. Physical examination revealed icterus and tender hepatomegaly. Radiological workup was planned on the same day, and high-resolution CT scan revealed ground-glass opacities throughout the lung fields, especially in the lower zones (Figure 1). Abdomen ultrasound revealed multiple, tubular echogenic structures within the proximal ileum with tapered ends and showing subtle movements with peristalsis, favoring the diagnosis of worms – most probably of the *Ascaris* species (Figure 2).

**Figure 3.**
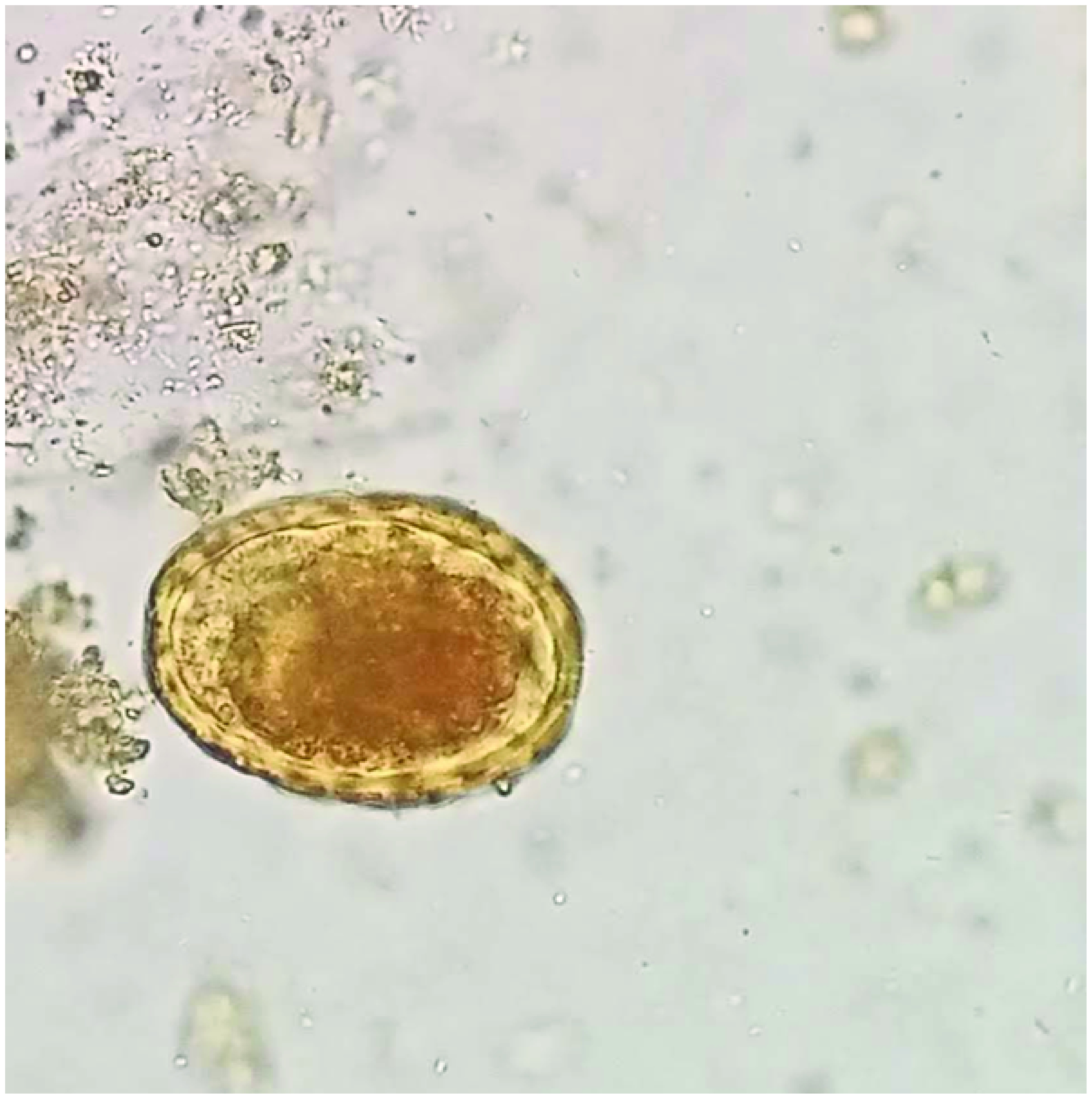
Stool microscopy revealing fertilized corticated eggs of *Ascaris lumbricoides*.

**Figure 4.**
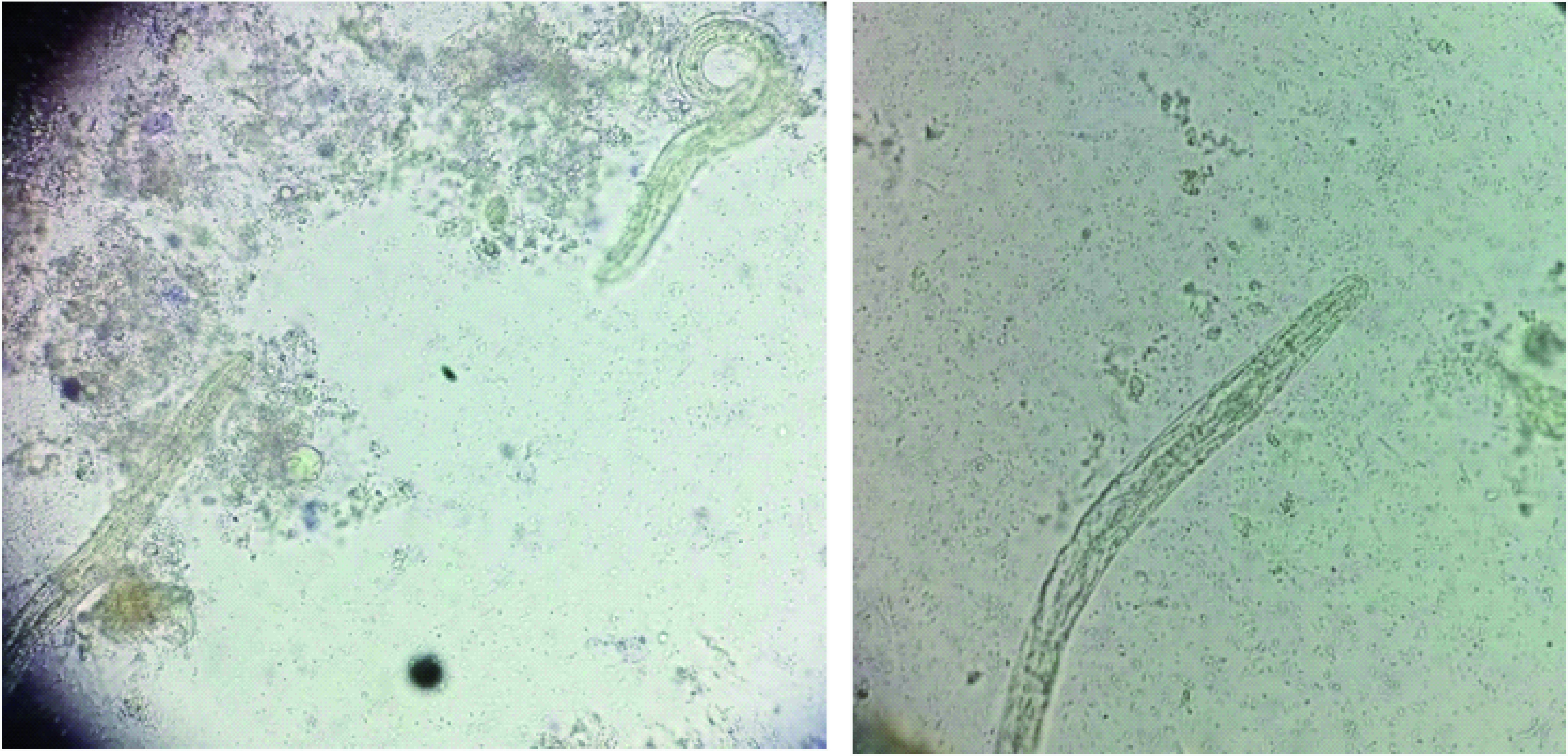
Stool sample showing rhabditiform larva (L1) of *Strongyloides stercoralis* on microscopic examination.

Direct stool examination was done on three sequential fresh stool specimens using a simple wet mount with saline and iodine. Microscopic examination under 40× lens revealed a number of fertilized corticated eggs/ova (45–60 μm in dimension) of *A. lumbricoides* (Figure 3). A large number of vermiform, elongated, worm/larvae-like structures were also observed in the stool sample (Figure 4). Detailed examination was done using Baermann-modified funnel and ethyl acetate concentration technique to reveal a number of fusiform worm-like structures. On examination under a high-resolution compound microscope, the worms/larvae were identified as rhabditiform larva (L1) of *S. stercoralis* based on the standard parasitological criteria [3]. The larvae were fusiform in shape, 241.8–249.6 μm in length and 15 μm in breadth; a double-bulbed esophagus with one corpus and one end bulb joined by a short isthmus, and a buccal cavity that was short, one-third to one-half as long as the width of the anterior end of the body along with a genital primordium at the posterior end. The stool sample was negative for any other cyst or trophozoites. Stool for occult blood was also negative. Kinyoun and trichrome staining for opportunistic pathogens was also negative.

Blood culture was also sent in a pair of BACTEC bottles (one aerobic, one anaerobic) to the microbiology laboratory for bacteriological workup. Routine blood culture was done before the initiation of antibiotics; the reports were sterile and did not reveal any significant abnormality or new microbial growth.

A new treatment regimen was initiated with hydroxychloroquine, favipiravir/remedesvir with azithromycin for COVID symptoms (as per treatment protocols as the case was seen in November 2021) and ivermectin (200 μg/kg orally daily) with oral albendazole (400 mg orally every 12 h) for presumed strongyloidiasis-*Ascariasis* coinfection [4].

Gradual resolution in respiratory as well as gastric symptoms were noticed in the patient with improvement in oxygen saturation. A second stool sample examination after 8 days was negative for any ova and parasite. He completed a 2-week course of ivermectin and albendazole as per treatment guidelines.

Repeat blood tests revealed slight improvement in biomarkers and blood counts. Repeat RT-PCR for COVID-19 at 16th day was negative, and CT scans also showed improvement in lung fields. The patient was discharged in good health on the 22nd day after admission with medications and outpatient visits.

## Discussion

The COVID pandemic has led to the emergence of various coinfections in the form of bacterial, fungal and parasitic colonizations in hospitalized patients [5–7]. SARS-CoV-2 influences the immune system of human beings both at cellular and humoral level. Helminthic parasites such as *S. stercoralis*, *A. lumbricoides*, *Trichuris* and hookworm which are known to be strongly associated with a Th2 cytokine shift accompanied by eosinophilia could represent a condition associated with rapid immunocompetence deterioration and their close association with COVID cases cannot be ruled out [8,9].

*Strongyloides* infection is usually seen as asymptomatic or a mild infection, but it can develop into severe disseminated strongyloidiasis in patients with immunosuppression. There have been several case reports of disseminated strongyloidosis already described in the COVID era [10–12]. The guidelines for screening and management of strongyloidiasis in endemic countries suggest that the cases should be screened with both direct (agar plate stool culture or Baermann technique) and indirect methods (serology); if there is a lack of adequate diagnostic facilities, empirical treatment with a single dose of ivermectin for 1 or 2 days is also recommended [13]. Ascariasis in adults also affects the immune system and leaves the body vulnerable to various infections and diseases [14]. COVID-19 is not an exempt in infectious diseases with immunosuppression as patient is already on steroid therapy. However, the possibility of a relationship between Ascaris and SARS-CoV-2 is still unexplored. Multiple helminth infections of different trematode, nematodes and cestode species are also commonly documented in previous studies. An infection with *S. stercoralis* was found to be positively associated with severe diabetes mellitus [15].

Use of steroids is one of the major risk factors associated with *Strongyloides* dissemination or hyperinfection syndrome because it inhibits the activation process of lymphocytes and eosinophils. The absolute eosinophil count also increased in the present case. The CT scans can distinguish between Löffler syndrome and COVID because COVID has a peculiar pattern presenting with ground-glass opacities, whereas Löffler syndrome has centrilobular pattern with micronodules in the lungs. Some studies have also reported increased fertility in females due to the use of these steroids. The exact mechanism behind this is unclear [16]. Our patient had been on oral steroids for rheumatoid arthritis for the previous 2 years, and this is an important factor for *Strongyloides–Ascaris* coinfection. The patient was a manual laborer with a relatively poor standard of living; the knowledge of health and hygienic practices can be correlated with the presence of coinfection of soil nematodes in such a case.

Radiologic imaging – in particular, abdominal ultrasound – is a good modality in patients presenting with acute abdomen. In the present case, ascariasis was very well diagnosed in the ileum as multiple, tubular echogenic structures within the proximal ileum having tapered ends and showing subtle movements with peristalsis. Stool examination in the microbiology laboratory proved conclusive in establishing the diagnosis of ascariasis by visualizing the fertilized corticated eggs of *A. lumbricoides*. Stool examination also proved useful in diagnosing coinfection with *S. stercoralis* by confirming the presence of rhabditiform larvae of *Strongyloides*. Treatment with antihelminthic drugs was effective in the eradication of the larvae/worms (repeat stool examination was negative) and complete clinical cure (symptom relief) of the patient. Albendazole is a preferred treatment option for a variety of helminthic infections such as *Ascaris*, pinworms and hookworms. Ivermectin has good action against *S. stercoralis*, whereas praziquantel is preferred for intestinal trematodes and nematodes [17]. The 2016 Committee to Advise on Tropical Medicine and Travel for Canada recommendations devised a guideline known as ‘test-and-treat’. This is for outpatient cases and for patients with mild COVID-19 symptoms (positive serology). Presumptive therapy with ivermectin may be considered for the cases of COVID-19 who are potential candidates for dexamethasone therapy and who may be at a higher risk for co-infection with *Strongyloides*. The role of preemptive treatment for strongyloides has a role in scenarios where serologic tests are not available or feasible for COVID and steroid therapy is required [18].

## Conclusion

This case report highlights the importance of parasitic coinfections in the COVID era. The present case is the first to identify the presence of ascariasis–strongyloidosis coinfection in a COVID patient in this part of the world (North India). Radiological and microbiological workup proved to be of immense help in the early diagnosis and prompt initiation of treatment. Detailed research work in this context will add further to the knowledge and understanding of these parasitic infections in the COVID scenario.

**Executive summary**

- Helminthic infestation is common among people living in tropical and subtropical areas. *Ascaris*, *Strongyloides*, *ancylostoma* and *trichuris*, among others, are some of the more common heminths worldwide.
- A 58-year-old male presented to the emergency department with chief complaints of breathlessness, dyspnea and acute colicky, periumbilical abdominal pain for the previous 4 days.
- The patient recalled the presence of fever, sore throat, dyspnea and gastric upset with upper respiratory tract symptoms from previous 14 days.
- Due to the ongoing COVID pandemic, he was advised tested; naso- and oropharyngeal swabs were obtained and sent for RT-PCR of COVID-19. The test was positive, and treatment was initiated with injectables including amphotericin B (for antifungal prophylaxis), aztreonam and oral methylprednisolone with random blood sugar monitoring.
- Eosinophil absolute count increased to >660 cells/ul on the fifth day of admission.
- Physical examination revealed icterus and tender hepatomegaly. Radiological workup was planned on the same day, and high-resolution CT scan revealed ground-glass opacities throughout the lung fields but especially in the lower zones.
- Abdominal ultrasound revealed multiple, tubular echogenic structures within the proximal ileum having tapered ends and showing subtle movements with peristalsis, favoring the diagnosis of worms – most probably *Ascaris* species.
- Direct stool examination was done on three sequential fresh stool specimens using a simple wet mount with saline and iodine. Microscopic examination under 40× lens revealed a number of fertilized corticated eggs/ova (45–60 μm in dimension) of *Ascaris lumbricoides* (Figure 3).
- A large number of vermiform, elongated, worm/larvae-like structures were also observed in the stool sample (Figure 4).
- Detailed examination was done using the Baermann-modified funnel and ethyl acetate concentration technique to reveal a number of fusiform worm-like structures. On examination under a high resolution compound microscope, the worms/larvae were identified as rhabditiform larva (L1) of *Strongyloides stercoralis* based on the standard parasitological criteria [3].
- A new treatment regimen was initiated with hydroxychloroquine, favipiravir/remedesvir with azithromycin for COVID symptoms and ivermectin (200 μg/kg orally daily) with oral albendazole (400 mg orally every 12 h) was for presumed strongyloidiasis-ascariasis coinfection.
- The COVID pandemic has led to the emergence of various coinfections in the form of bacterial, fungal and parasitic colonizations in hospitalized patients.
- Use of steroids is one of the major and important risk factors associated with *Strongyloides* dissemination or hyperinfection syndrome as it inhibits the activation process of lymphocytes and eosinophils.
- The present case is the first to identify the presence of ascariasis-strongyloidosis coinfection in COVID patient in North India.
- Combined radiological and microbiological workup help in the early diagnosis and prompt initiation of treatment in the patient.
- Detailed research work will add further to the knowledge and understanding of these parasitic infections in the COVID scenario.
